# Midwifery Continuity of Care During Pregnancy, Birth, and the Postpartum Period: A Matched Cohort Study

**DOI:** 10.1111/birt.12875

**Published:** 2024-10-28

**Authors:** L. Lundborg, K. Åberg, X. Liu, M. Norman, O. Stephansson, K. Pettersson, M. Ekborn, S. Cnattingius, M. Ahlberg

**Affiliations:** ^1^ Clinical Epidemiology Division, Department of Medicine, Solna Karolinska Institutet Stockholm Sweden; ^2^ Department of Clinical Science, Intervention, and Technology Karolinska Institutet Stockholm Sweden; ^3^ Department of Neonatal Medicine Karolinska University Hospital Stockholm Sweden; ^4^ The Swedish Neonatal Quality Register Stockholm Sweden; ^5^ Department of Women's Health Karolinska University Hospital Stockholm Sweden

**Keywords:** birth outcomes, continuity of care, medical interventions

## Abstract

**Objective:**

To compare pregnancy outcomes in a midwifery continuity of care (MCoC) model to standard midwifery care in Sweden.

**Design:**

Matched cohort study.

**Setting:**

Public healthcare during pregnancy and childbirth, Stockholm, Sweden.

**Population:**

Women giving birth at Karolinska University Hospital site Huddinge in Stockholm between January 1, 2019, and August 31, 2021.

**Methods:**

Data on all births including MCoC and standard care, during the time period, were retrieved from the national Swedish Pregnancy Register. Propensity score matching was applied to obtain a matched set from the standard care group for every woman in the MCoC model. Based on the matched cohort, we estimated risk ratios (RR) for binary outcomes with 95% confidence intervals (CI).

**Main Outcome Measures:**

Interventions during labor, mode of birth, and preterm birth (< 37 gestational weeks).

**Results:**

Compared with standard care, women in the MCoC model were more likely to give birth spontaneously (RR 1.06 95% CI 1.02–1.10) and less likely to have an elective cesarean on maternal request (RR 0.24 95% CI 0.11–0.51). The risk of preterm birth was also reduced in the MCoC group (RR 0.51 95% CI 0.32–0.82).

**Conclusion:**

The MCoC model was associated with fewer medical interventions and improved pregnancy outcomes.

## Graphical abstract

This matched cohort study showed that Midwifery Continuity of Care was associated with lower risk of medical interventions and preterm birth with no increased risks of adverse outcomes.

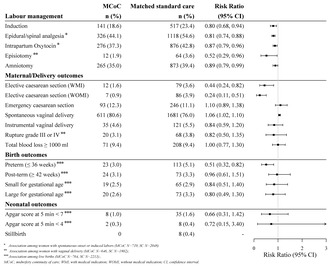

## 1 Introduction

Midwifery continuity of care (MCoC) models include monitoring of physical, psychological, and social well‐being of pregnant persons and their families by providing individualized, relational, ongoing support during pregnancy, birth, and postpartum period.

There is strong evidence supporting the beneficial effects of MCoC models on pregnancy‐related outcomes. A meta‐analysis of 17 randomized controlled trials concluded that MCoC increases spontaneous vaginal births, increases satisfaction with care, and reduces the need for medical interventions compared with standard care [1]. Furthermore, observational studies suggest that MCoC is associated with a reduction in cesarean births, induction of labor, and perineal trauma [2, 3, 4]. In MCoC models, birthing people report feeling safer and trusting of their care providers [5, 6], more confident, and less stressed compared to standard care [7].

In 2018, an MCoC model was implemented at Karolinska University Hospital (KUH), Stockholm, alongside standard care. The aim of the model was to offer women with fear of birth additional support to give birth vaginally. The MCoC model at KUH is the first complete MCoC model implemented in Sweden.

A scientific validation plan for the project was launched to evaluate overall safety in the MCoC model and to examine whether the MCoC model was associated with improved outcomes by using data from the Swedish Pregnancy Register.

The aim of this study was to compare pregnancy outcomes among women receiving care within the MCoC model with a matched group of pregnant women in standard care. We investigated traditional medical performance indicators, such as number of medical interventions, number of spontaneous births, preterm birth, and number of healthy newborns.

## 2 Methods

### 2.1 Population

**FIGURE 1:**
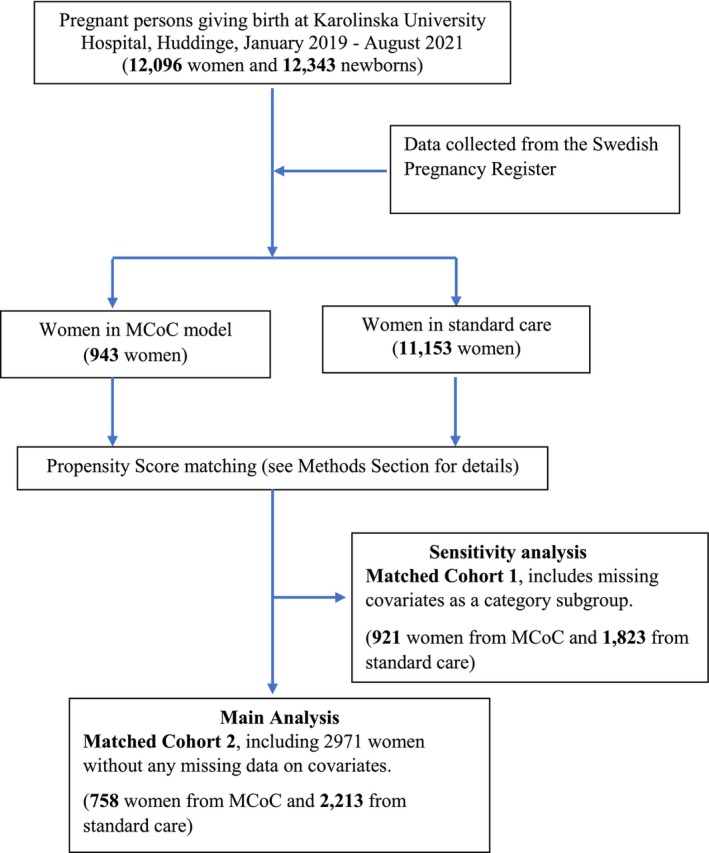
Flowchart of two matched cohorts using different methods to handle missing covariate data. Results from the analysis on Matched Cohort 2 are mainly presented and discussed, with comparisons to results from analyses on Matched Cohort 1 in Appendix S1 (treated missing covariates as a category–subgroup, respectively). MCoC, midwifery continuity of care. [Colour figure can be viewed at wileyonlinelibrary.com]

The study population included all women giving birth at KUH from January 1, 2019, to August 31, 2021. KUH is a university hospital with approximately 4000 births every year. This site offers delivery care from gestational Week 24 to all women. All women in Sweden have access to public healthcare that is government funded and free of charge. Figure 1 describes the population sampling process.

### 2.2 Setting

#### 2.2.1 Standard Antenatal Care (SAC)

Maternity care providers are predominantly midwives in primary antenatal care, with consultation with a specialist at the hospital when complications occur. A common system exists for women who need specialist consultations within hospital settings, for example, Type 1 diabetes, fear of childbirth, or other chronic conditions that require additional medical attention according to guidelines. Women with medical conditions will continue their regular pregnancy check‐ups with their midwives in SAC in combination with specialist consultations at the hospital. SAC includes a minimum of eight to nine appointments during pregnancy and ultrasound for pregnancy dating at 18–20 weeks gestation. SAC strives to keep some continuity of caregiver, but individual differences exist and most women meet a minimum of two different midwives during pregnancy [8]. The SAC model in Stockholm is accessible for women during business hours on weekdays and does not include home visits pre‐ or postpartum. Any concern during pregnancy that arises after office hours or on weekends is referred to the hospital delivery suite.

Women in SAC give birth in a hospital where they are cared for by other staff than those working at the antenatal clinic. Information about the woman's health status before and during pregnancy is shared through electronic health records. The hospital is responsible for the woman and newborn until 7 days postpartum, thereafter the woman is re‐referred to SAC for standard check‐ups at 6–8 weeks postpartum or according to individual needs. The responsibility for the newborn is referred to community‐based child clinics 7 days postpartum. Women with fear of birth are either offered extra consultations with the midwife in SAC or are referred to specialized units at the hospital for consultations for fear of birth. Women with fear of birth but without medical complications are encouraged to opt for a vaginal birth during the consultations.

### 2.3 MCoC Model

The MCoC model started in September 2018 with two teams of three midwives each. The model successively expanded, and in September 2020, four teams with four midwives on each team were in operation. Each midwife cared for approximately 40 women per year.

The MCoC teams were organized to enable relational continuity for the pregnant woman with the midwives throughout pregnancy, birth, and postpartum. The teams offered primary antenatal care using the same guidelines, referral systems to specialist care, and number of minimum visits as SAC. All MCoC teams were placed in already existing primary antenatal clinics working alongside midwives in SAC. The MCoC model offers, as opposed to SAC, continuity of care, home visits in early labor and postnatally, and on‐call consultations around the clock. Homebirths have been offered within the model since October 2020 with a capacity to manage a total of 40 homebirths per year. Postpartum care is offered up to 7 days postpartum at home and one or two scheduled meetings at the antenatal clinic at approximately 6–8 weeks postpartum.

All women cared for in the MCoC model were self‐referred in early pregnancy by making a request online or by telephone. The MCoC model was open for all women regardless of medical risk level and priority was given to women with fear of birth. The MCoC midwives use the same system for specialist consultations as in SAC caring for women with medical conditions. A midwife from the MCoC team called all women who expressed interest and informed them about the model. Fear of birth was not quantified, and there was no cut‐off for participation; any woman who expressed any level of fear of birth was accepted if they were willing to aim for a vaginal birth and to give birth at KUH. Women, in early pregnancy, who desired an elective cesarean birth, declined treatment for fear of birth and refused more discussion around mode of delivery were not enrolled.

All women with fear of birth were counseled by their midwives within the model and were not referred to the specialized unit at the hospital. Women were offered extra consultations to talk about their fear of birth. All women in the model had access to elective cesarean without medical indication if this was what they ended up opting for in late pregnancy.

### 2.4 Data Collection

Data on all births at the Karolinska University Hospital site Huddinge from January 1, 2019, to August 31, 2021, were retrieved. Information on maternal characteristics, including maternal medical history, and antenatal and obstetric care from pregnancy throughout birth and postpartum, was collected. Patients were not involved in the development of the research.

### 2.5 Design and Data Sources

This matched cohort study used data from the Swedish Pregnancy Register (SPR), a national quality register that contains prospectively collected data from routinely used electronic antenatal, obstetric, postpartum, and postnatal records from 22 completed gestational weeks in Sweden. The register contains information on all deliveries (100%) in the Stockholm Region. Data from the standardized electronic medical records are automatically forwarded to the SPR after birth, with linkage to the unique personal registration number of mothers and infants [9].

### 2.6 Exposure and Control

Women cared for in the MCoC model during pregnancy comprised the exposure group. All midwives had a personal identification code available in the SPR, and the MCoC midwives only cared for pregnant women admitted to the model. All women receiving community‐based antenatal standard care during pregnancy and who gave birth at KUH during the same time period comprised the control or unexposed group.

### 2.7 Pregnancy Outcomes

Maternal outcomes included: mode of birth (spontaneous vaginal birth, instrumental vaginal birth, elective cesarean birth with or without a medical indication [i.e., on maternal request]), emergency cesarean, perineal rupture grade III/IV, and postpartum hemorrhage (≥ 1000 mL). Outcomes related to labor interventions included: induction of labor, amniotomy, oxytocin for labor augmentation, epidural/spinal analgesia, and episiotomy.

The following birth outcomes were assessed: preterm and postterm birth (< 37+0 and ≥ 42+0 gestational weeks), both based on embryo transfer or standard ultrasound dating of pregnancy or, if ultrasound dating was missing, based on last menstrual period; small and large for gestational age (SGA and LGA, defined as < 2SD and > 2SD from mean weights for gestational age, respectively, according to the sex‐specific Swedish fetal growth curve) [10]. Some outcomes related to labor management were assessed using ICD‐10 diagnostic codes and procedure codes (Table S1).

### 2.8 Covariates

Information on maternal characteristics included maternal age at delivery, height, parity, and body mass index (BMI in kg/m^2^) at first antenatal visit, multiple pregnancies, prepregnancy comorbidity, previous or ongoing psychiatric care, previous cesarean birth, daily smoking at first antenatal visit, number of years of formal education, mother's birth region, and giving birth during COVID‐19 pandemic (from March 2020 onwards). Variables were categorized according to Table S2.

### 2.9 Statistical Analysis

#### 2.9.1 Propensity Scores and Matching

First, we calculated descriptive statistics of maternal characteristics and outcomes in MCoC and standard care groups (Tables 1 and 2, respectively). Then, we calculated a propensity score for every woman through a logistic regression model on selected covariates presented in Tables 1 and 3. The covariates were related to the distribution of outcomes between MCoC and standard care groups before matching [11]. The propensity score matching aimed to remove the effects of confounding from these covariates (Tables 1 and 3) and mimic some characteristics of a randomized controlled trial. We applied the nearest‐neighbor matching algorithm on the logit of the propensity score without replacement, using a maximum caliper width of 0.2 of its standard deviation and a matching ratio of 1:3 in complete case analysis (Matched Cohort 2 in Figure 1).

**TABLE 1:** Descriptive statistics of covariates (before matching) among women who gave birth at Karolinska University Hospital, Huddinge, Sweden, from January 1, 2019, to August 31, 2021.

Characteristic, *N* (%)	MCoC	Standard care
Pregnant persons	943	11,153
Newborn infants	950	11,393
Maternal age, mean (SD), years	33.5 (3.8)	31.8 (5.1)
15–24	10 (1.1)	1003 (9.0)
25–29	160 (17.0)	3127 (28.0)
30–34	451 (47.8)	4043 (36.3)
≥ 35	322 (34.1)	2980 (26.7)
Maternal height, mean (SD), cm	167.8 (6.6)	164.6 (6.7)
≤ 159	83 (9.0)	2362 (21.6)
160–164	210 (22.7)	3066 (28.0)
165–169	254 (27.4)	2839 (25.9)
≥ 170	380 (41.0)	2678 (24.5)
Missing	16	208
Parity		
Primiparity	522 (55.4)	4584 (41.1)
Multiparity	421 (44.6)	6568 (58.9)
Missing	0	1
Early pregnancy BMI, mean (SD), kg/m^2^	24.0 (4.1)	25.6 (5.0)
Underweight (< 18.5)	17 (1.9)	229 (2.1)
Normal weight (18.5–24.9)	597 (66.3)	5461 (50.9)
Overweight (25.0–29.9)	209 (23.2)	3175 (29.6)
Obese (≥ 30.0)	78 (8.7)	1863 (17.4)
Missing	42	425
Multiple pregnancies	7 (0.7)	237 (2.1)
Prepregnancy comorbidity^a^		
Yes	433 (46.9)	3805 (34.9)
No	490 (53.1)	7090 (65.1)
Missing	20	258
Any psychiatric care		
Yes	318 (34.7)	1835 (16.9)
No	598 (65.3)	9007 (83.1)
Missing	27	311
Previous Cesarean	50 (5.3)	1502 (13.5)
Smoking status at first antenatal visit		
Smoker	7 (0.8)	435 (4.0)
Nonsmoker	850 (99.2)	10,445 (96.0)
Missing	86	273
Level of education, years		
≤ 9	5 (0.6)	977 (10.1)
10–12	116 (13.0)	3567 (36.8)
> 12	769 (86.4)	5137 (53.1)
Missing	53	1472
Mother's birth country		
Nordic	801 (86.1)	5308 (51.7)
European (non‐Nordic)	52 (5.6)	1129 (11.0)
Middle East/African	26 (2.8)	2280 (22.2)
Others	51 (5.5)	1554 (15.1)
Missing	13	882
Given birth during COVID‐19 period (since 2020 March)	654 (69.4)	6356 (57.0)

Abbreviation: MCoC, midwifery continuity of care.

^a^ Prepregnancy comorbidity, including cardiovascular disease, liver disease, diabetes, gynecological disease, lung disease, endocrine disease, kidney disease, inflammatory bowel disease, chronic hypertension, and neurological disorder.

**TABLE 2:** Descriptive statistics of outcomes (before matching) among women who gave birth at the Karolinska University Hospital Huddinge, Sweden, from January 1, 2019, to August 31, 2021.

Outcome, *N* (%)	MCoC	Standard care (SC)
Pregnant women	943	11,153
Newborn infants	950	11,393
Maternal outcomes		
Mode of birth		
Elective cesarean (WMI)	14 (1.5)	548 (4.9)
Elective cesarean (WOMI)	8 (0.8)	489 (4.4)
Emergency cesarean	111 (11.8)	1515 (13.6)
Spontaneous vaginal	763 (80.9)	8146 (73.0)
Instrumental vaginal	47 (5.0)	455 (4.1)
Rupture grade III or IV^a^	25 (3.1)	311 (3.6)
Total blood loss ≥ 1000 mL		
Yes	89 (9.5)	1129 (10.2)
No	851 (90.5)	9990 (89.8)
Missing	3	34
Labor management		
Induction of labor	187 (19.8)	2706 (24.3)
Regional analgesia^b^	411 (44.6)	5328 (52.7)
Intrapartum oxytocin^b^	345 (37.5)	4021 (39.7)
Episiotomy^a^	15 (1.9)	214 (2.5)
Amniotomy	327 (34.7)	4624 (41.5)
Birth outcomes		
Gestational age, days		
Mean (SD)	278.9 (11.9)	275.2 (15.4)
Min, Max	165, 297	154, 300
Missing	0	3
Gestational age, completed weeks^c^		
22–27	5 (0.5)	66 (0.6)
28–31	2 (0.2)	123 (1.1)
32–36	21 (2.2)	671 (5.9)
37–41	892 (93.9)	10,071 (88.8)
≥ 42	30 (3.2)	430 (3.8)
Missing	0	3
Birthweight for gestational age^c^		
SGA	24 (2.5)	417 (3.7)
AGA	903 (95.2)	10,511 (92.9)
LGA	22 (2.3)	382 (3.4)
Missing	1	34
Apgar score at 5 min^c^		
0–6	14 (1.5)	192 (1.7)
7–10	933 (98.5)	11,095 (98.3)
Missing	3	57
Apgar score at 5 min < 4^c^		
Yes	4 (0.4)	38 (0.3)
No	943 (99.6)	11,249 (99.7)
Stillbirth	0	46 (0.4)
Missing	0	3

Abbreviations: AGA, appropriate for gestational age (−2 to 2SD); LGA, large for gestational age (> 2 SD); MCoC, midwifery continuity of care; SGA, small for gestational age (< −2 SD); WMI, with medical indications; WOMI, without medical indications.

^a^ Summary among women with vaginal deliveries (MCoC *N* = 810, SC *N* = 8601).

^b^ Summary among spontaneous onset and induced labors (MCoC *N* = 921, SC *N* = 10,116).

^c^ Summary among live births (MCoC *N* = 950, SC *N* = 11,344).

**TABLE 3:** Matched Cohort 2. Covariate balance (after matching^a^) across MCoC and standard care groups, indicated by absolute standardized difference.

Characteristic	MCoC (758 women)	Matched standard care (2213 women)	Absolute standardized difference (< 0.1)
Age, years, mean (SD)	33.5 (3.8)	33.3 (4.1)	0.032
15–24	10 (1.3%)	21 (0.9%)	0.049
25–29	129 (17.0%)	378 (17.1%)	
30–34	363 (47.9%)	1097 (49.6%)	
≥ 35	256 (33.8%)	717 (32.4%)	
Height, cm, mean (SD)	167.8 (6.6)	167.7 (6.2)	0.028
≤ 159	66 (8.7%)	176 (8.0%)	0.036
160–164	171 (22.6%)	525 (23.7%)	
165–169	210 (27.7%)	615 (27.8%)	
≥ 170	311 (41.0%)	897 (40.5%)	
BMI, kg/m^2^, mean (SD)	24.0 (4.1)	24.2 (4.0)	0.046
Underweight	16 (2.1%)	39 (1.8%)	0.055
Normal weight	501 (66.1%)	1455 (65.7%)	
Overweight	176 (23.2%)	551 (24.9%)	
Obese	65 (8.6%)	168 (7.6%)	
Parity			
Primiparity	420 (55.4%)	1168 (52.8%)	0.053
Multiparity	338 (44.6%)	1045 (47.2%)	
Multiple pregnancies	6 (0.8%)	8 (0.4%)	0.057
Any psychiatric care	249 (32.8%)	652 (29.5%)	0.073
Level of education, years			
≤ 9	4 (0.5%)	17 (0.8%)	0.034
10–12	95 (12.5%)	289 (13.1%)	
> 12	659 (86.9%)	1907 (86.2%)	
Mother's birth country			
Nordic	653 (86.1%)	1935 (87.4%)	0.043
Europe (non‐Nordic)	46 (6.1%)	127 (5.7%)	
Middle East/Africa	21 (2.8%)	58 (2.6%)	
Others	38 (5.0%)	93 (4.2%)	
Given birth during COVID‐19 period (since 202003)	545 (71.9%)	1560 (70.5%)	0.031
Prepregnancy comorbidity	348 (45.9%)	971 (43.9%)	0.041
Smoking status at first antenatal care	6 (0.8%)	16 (0.7%)	0.008
Previous cesarean	33 (4.4%)	112 (5.1%)	0.033

Abbreviation: MCoC, midwifery continuity of care.

^a^ Age, height, and BMI in categorical forms used to estimate propensity score.

After propensity score matching, covariate balance was checked across exposed and nonexposed groups (statistics presented in Table 3). Absolute standardized difference was calculated separately for continuous, binary, and categorical variables and evaluated as having good balance according to the criterion of absolute standardized difference < 10%.

#### 2.9.2 Main and Sensitivity Analyses

Based on the matched cohort (Matched Cohort 2), we used modified Poisson regressions to estimate risk ratios (RR) with 95% confidence intervals (CI) for binary outcomes [12, 13]. Robust sandwich‐type variance was estimated to calculate 95% CIs for RRs. Two sensitivity analyses were performed to assess the robustness of our results. First, the ratio of exposed‐to‐nonexposed women was explored from 1:1 to 1:2 to 1:3 until covariate balance was reduced (controlled by absolute standardized difference < 10%); second, we applied two methods to handle missing covariates in propensity score analyses, missing indicator method versus complete case analysis, with results presented in Tables S2 and S3.

Analyses were performed in SAS version 9.4 (SAS Institute Inc., Cary, NC, USA) and R version 4.1.2 (R Core Team, 2021; R Foundation for Statistical Computing, Vienna, Austria), with the MatchIt, stddiff, and geeglm packages.

## 3 Results

A total of 12,096 women gave birth at Karolinska University Hospital site Huddinge during the time period, of whom 921 women were identified as being cared for in the MCoC model.

### 3.1 Study Characteristics Before Propensity Score Matching

Compared with women in standard care, women in the MCoC model were generally older, taller, had lower BMIs, and a higher level of education, and were often nonsmokers (Table 1). Women in the MCoC model were also more often primiparous, born in the Nordic Region of Europe, and more likely to have prepregnancy comorbidities and psychiatric disorders noted in the medical record. Furthermore, a higher proportion of women in the MCoC model gave birth during the COVID‐19 period (from March 2020) compared to the control group (69.4% vs. 57%).

Birth outcomes before matching showed that preterm births were rare in both models, although lower in the MCoC model. The rates of postterm pregnancies were similar between the models of care.

All outcomes related to labor management (labor induction, regional analgesia, oxytocin use, episiotomy, and amniotomy) were lower in the MCoC model compared with the standard model. The proportions of infants with Apgar score < 7 at 5 min were similar between the two models. There were no stillbirths in offspring of mothers in the MCoC model, and a total of 46 stillbirths (0.4%) occurred in offspring of mothers receiving standard care (Table 2).

### 3.2 Main Analysis and Propensity Score‐Matched Results

After propensity score 1:3 matching, we identified 758 women in the MCoC model and 2213 standard care controls with full information on the matching variables (Matched Cohort 2). After matching, the mean values for maternal age, height, and BMI and the proportions of categorized covariates were similar in the two groups (Table 3).

### 3.3 Labor Onset and Labor Management

After matching, compared with standard care, women in the MCoC model were at 20% lower risk of having an induction of labor. Furthermore, women in the MCoC model were less likely to experience labor interventions, such as amniotomy, labor augmentation with artificial oxytocin, and episiotomy (Table 4).

**TABLE 4:** Maternal and neonatal outcomes in MCoC versus standard care, with association measures by risk ratios and 95% confidence intervals.

Outcomes	MCoC (758 women)	Matched standard care (2213 women)	Risk ratio (95% CI)
Labor management, *N* (%)			
Induction	141 (18.6%)	517 (23.4%)	0.80 (0.68, 0.94)
Epidural/spinal analgesia^a^	326 (44.1%)	1118 (54.6%)	0.81 (0.74, 0.88)
Intrapartum oxytocin^a^	276 (37.3%)	876 (42.8%)	0.87 (0.79, 0.96)
Episiotomy^b^	12 (1.9%)	64 (3.6%)	0.52 (0.29, 0.96)
Amniotomy	265 (35.0%)	873 (39.4%)	0.89 (0.79, 0.99)
Maternal and delivery outcomes, *N* (%)			
Elective cesarean (WMI)	12 (1.6%)	79 (3.6%)	0.44 (0.24,0.82)
Elective cesarean (WOMI)	7 (0.9%)	86 (3.9%)	0.24 (0.11,0.51)
Emergency cesarean	93 (12.3%)	246 (11.1%)	1.10 (0.89, 1.38)
Spontaneous vaginal delivery	611 (80.6%)	1681 (76.0%)	1.06 (1.02, 1.10)
Instrumental vaginal delivery	35 (4.6%)	121 (5.5%)	0.84 (0.59, 1.20)
Rupture Grade III or IV^b^	20 (3.1%)	68 (3.8%)	0.82 (0.50, 1.35)
Total blood loss ≥ 1000 mL	71 (9.4%)	208 (9.4%)	1.00 (0.77, 1.30)
Birth outcomes, *N* (%)			
Preterm (≤ 36 weeks)^c^	23 (3.0%)	113 (5.1%)	0.51 (0.32, 0.82)
Postterm (≥ 42 weeks)^c^	24 (3.1%)	73 (3.3%)	0.96 (0.61, 1.51)
Small for gestational age^c^	19 (2.5%)	65 (2.9%)	0.84 (0.51, 1.40)
Large for gestational age^c^	20 (2.6%)	73 (3.3%)	0.80 (0.49, 1.30)
Neonatal outcomes, *N* (%)			
Apgar at 5 min < 7^c^	8 (1.0%)	35 (1.6%)	0.66 (0.31, 1.42)
Apgar at 5 min < 4^c^	2 (0.3%)	8 (0.4%)	0.72 (0.15, 3.40)
Stillbirth	0	8 (0.4%)	—

Abbreviations: MCoC, midwifery continuity of care; WMI, with medical indications; WOMI, without medical indications.

^a^ Association among women with spontaneous onset or induced labor (MCoC *N* = 739, SC *N* = 2048).

^b^ Association among women with vaginal delivery (MCoC *N* = 646, SC *N* = 1802).

^c^ Association among live births (MCoC *N* = 764, SC *N* = 2213).

### 3.4 Pregnancy Outcomes

After matching, compared with standard care, women in the MCoC model had a more than 50% risk reduction for elective cesarean regardless of indication and were slightly more likely to have a spontaneous vaginal delivery (Table 4). Emergency cesareans was similar between the groups. No difference was found between the groups regarding instrumental vaginal delivery, Rupture Grade III or IV, or postpartum hemorrhage.

Compared with standard care, after matching, women in the MCoC model had a 49% relative risk reduction of preterm birth (< 37 weeks). Sensitivity analysis showed that the risk for spontaneous preterm birth was substantially decreased in the MCoC model (RR 0.42 95% CI 0.25–0.71), while there was no difference between the two models of care in the risk of medically indicated preterm birth (Table 5). The risk of preterm birth was further estimated with stratification for parity. For nulliparous women, the reduced risk in the MCoC model remained (RR 0.51 95% CI 0.29–0.88), while no significant differences were observed between the two groups of care for parous women (RR 0.58 95% CI 0.26–1.28; Table S4).

**TABLE 5:** Summary of associations of preterm birth with MCoC among women with either spontaneous start or labor induction.

Outcomes	MCoC	Matched standard care	Risk ratio (95% CI)
Complete case analysis, excluding stillbirth and elective cesarean			
Preterm birth, *spontaneous start*	19 (3.2)	96 (6.2)	0.42 (0.25, 0.71)
Preterm birth, *labor induction*	3 (2.1)	13 (2.5)	0.82 (0.24, 2.85)

Abbreviation: MCoC, midwifery continuity of care.

No differences were observed between the two models of care after matching on Apgar score < 7 or < 4 at 5 min of age. There were no stillbirths in the MCoC model and 8 stillbirths in the matched cohort (Table 4).

## 4 Discussion

### 4.1 Main Findings

This cohort study using data from the Swedish Pregnancy Register showed that an MCoC model was associated with a reduction in medical interventions and improved pregnancy outcomes compared to a matched control group receiving standard care. The most striking findings were the lower rate of preterm birth and the reduction in elective cesarean birth in the MCoC model compared to the standard model. No investigated outcomes favored standard care.

### 4.2 Strengths and Limitations

A major strength of this study was our ability to use prospectively collected, real‐world data directly forwarded to the Pregnancy Register from the electronic medical records. Doing so eliminated the risk of loss to follow‐up. By selecting a matched comparison group giving birth at the same hospital as the exposed group, we minimized confounding caused by different clinical guidelines, staffing levels, or competence at different hospitals providing care in Stockholm. The propensity scores‐matched design of this study enabled us to balance factors known to affect the risk of preterm birth across the groups, which was essential after observing differences in the risk factors for preterm birth between MCoC and SAC. Our results showed robustness in all performed sensitivity analyses. Propensity score matching was used to balance known risk factors between the groups, nevertheless, some residual confounding cannot be ruled out with this study design.

There are several limitations to this study. It is an observational study and results must be interpreted cautiously. Still, the results are in line with previous results from several RCTs, and some of the risk reductions found in this study were large. As randomized clinical trials may face practical challenges such as a high proportion of patients who decline participation, individual benefit, and intervention use patterns that differ from real‐world application [14], population‐based longitudinal cohorts or national registers are important for perinatal science, especially for research involving rare perinatal outcomes [15].

The matched comparison group was chosen among birthing people at the same hospital (KSH) to eliminate factors such as hospital‐specific guidelines and staffing levels that might influence outcomes. However, this decision might influence the generalizability of the findings if MCoC care is implemented in different settings or hospitals. Larger population‐based studies including several sites and hospitals are warranted.

A major limitation is that we lacked information about fear of birth in the register. Local questionnaires were used among women admitted to the MCoC model showing a prevalence of 70% with fear of birth (any level), but we lack information about the prevalence of fear of birth in the group of women receiving standard care. Swedish estimates of fear of birth among pregnant women range from 14% to 20% [16, 17].

Due to lack of information on fear of birth, we were not able to investigate whether fear of birth differences between the MCoC and SAC groups influenced birth outcomes. Furthermore, women who in early pregnancy were unwilling to receive treatment for fear of birth and discuss mode of delivery were referred to standard care for treatment for fear of birth, indicating a selection bias in the MCoC group. A Swedish study published in 2012 found that women with fear of birth who received treatment for fear of birth had a cesarean rate of 29.5% [18]. Another Swedish study showed that among women who underwent counseling, fear of childbirth was associated with a three to six times higher rate of elective cesarean with a prevalence of 29.8% among women with fear of birth [19]. The prevalence of elective cesarean before matching was 2.3% and after matching was 2.5% among women treated in MCoC. Our findings suggest a very large reduction in elective cesarean births in this study as compared to previous results from SAC.

A notable limitation of using population‐based data is the lack of input from the women themselves.

### 4.3 Interpretations

This is the first validation of the Karolinska MCoC model, a full‐scale hospital‐integrated continuity of care model in a Swedish context using data from the Swedish Pregnancy Register. Focusing on all traditional performance indicators, results indicate that the MCoC model is a safe and favorable option compared to standard care in Swedish settings. Women within the MCoC model received fewer medical interventions and experienced a reduced risk of many adverse pregnancy outcomes. Our results are in line with results from the meta‐analysis by Sandall et al., for example, lower rates of cesarean birth, epidural analgesia, episiotomies, and an increase in spontaneous vaginal birth [1]. We report a significant reduction in preterm birth which was not found in the latest updated review from 2024 [1]. In this study, the rate of preterm birth was slightly higher in the unmatched control group compared to the national rate (7.6% vs. 5.8%, respectively) [20]. The overall higher prevalence of preterm birth at KUH compared to overall data from Sweden is to be expected, as this hospital's geographical area has a higher prevalence of families with low socio‐economic status, foreign‐born women, and women with obesity, all risk factors for preterm birth [21].

Several additional methods were used to test the robustness of the findings on preterm birth. Firstly, to test if the decreased risk of preterm birth was due to iatrogenic factors, we stratified on labor onset and found that the increased risk was restricted to spontaneous preterm births (Table 5). These findings are important since stress and pregnancy‐related anxiety have been shown to increase the risk of spontaneous preterm birth [22]. Reduced levels of stress and anxiety is a plausible pathway for how the MCoC model can reduce the prevalence of preterm birth [1, 22, 23].

The finding that the MCOC model was associated with a substantially reduced risk of elective cesarean without a medical indication (maternal request) is novel. Previous studies have shown that MCoC can reduce elective cesareans, but information on indication for the cesareans is lacking [1, 24, 25]. From 1990 to 2015, the prevalence of cesarean births on maternal request increased from 0.6% to 4.6% in Sweden [26]. Although several efforts to support women to opt for a vaginal birth have been made, evidence of effective treatment and care is lacking [27]. A review reported that fear of birth, anxiety, and fear of loss of control were major reasons why women requested an elective cesarean [28]. MCoC models have been shown to increase feelings of safety and sense of control and to reduce anxiety, and these are influencing women's plans for how to give birth [29, 30, 31]. Some of the women in the MCoC were supported in their decision‐making to opt for a vaginal birth, and this may have influenced the elective cesarean delivery rate in this study.

That the MCoC model was associated with more spontaneous vaginal births and fewer medical interventions without jeopardizing medical safety is important. Emerging evidence shows that spontaneous vaginal births with few medical interventions are associated with better short‐ and long‐term outcomes for women and their infants [32, 33]. Fewer women in the MCoC model required induction of labor, regional anesthesia, augmentation with oxytocin, amniotomy, and episiotomy.

A difference between the two models of care that might influence both interventions during labor and birth outcomes is that the MCoC model provides one‐to‐one care during labor. In standard care, one‐to‐one care cannot be guaranteed due to staffing levels. Benefits described in randomized trials investigating one‐to‐one care include decreased need for analgesia, fewer operative deliveries, and fewer newborns with low 5 min Apgar score [34, 35].

## 5 Conclusion

We present data from the first study validating a hospital‐integrated midwifery continuity of care model targeting women with fear of birth in a Swedish setting. Information about exposure, outcomes, confounders, and mediators was collected from the Swedish Pregnancy Register. All investigated variables support the implementation of the MCoC model as a safe and effective way to improve pregnancy outcomes. MCoC was associated with a lower preterm birth rate, fewer medical interventions, and improved neonatal and maternal outcomes compared with standard care, and no adverse effects were found. Results should inform policy making that MCoC could be one way to improve care for women with fear of birth.

## Ethics Statement

The National Ethical Committee, situated in Uppsala, Sweden, approved the study protocol (Reference No 2021–02722). Date of approval 20‐05‐2021. The database is stored in the Unit of Clinical Epidemiology at Karolinska Institutet Stockholm, Sweden. Public data sharing from this database is not permitted. Any questions regarding public access to the data are handled by the Division of Clinical Epidemiology. Department of Medicine Solna, Karolinska Institutet.

## Conflicts of Interest

The authors declare no conflicts of interest.

## Supporting information


Appendix S1.

## Data Availability

The data that support the findings of this study are available in Graviditetsregistret at https://www.medscinet.com/gr/default.aspx. These data were derived from the following resources available in the public domain: Graviditetsregistret, https://www.medscinet.com/gr/default.aspx.
