# Supplementary material for: Midwifery Continuity of Care During Pregnancy, Birth, and the Postpartum Period: A Matched Cohort Study
Source: Birth. 2024 Oct 28;52(1):146–56. doi: 10.1111/birt.12875 (PMC11829270; doi:10.1111/birt.12875)
Supplement: Supplementary file 1 — Appendix S1. [file BIRT-52-146-s001.docx]

**Supplementary materials for Midwifery Continuity of Care during Pregnancy, Birth and the Postpartum period: a matched cohort study.**

**S. Table 1.**

Diagnostic codes used in this study to identify cases of outcomes.

**S. Table 2 (Results from analyses on matched cohort 1).**

Checking on covariate balance (after matching *) across MCoC and standard care groups, indicated by absolute standardized difference (<0.1).

**S. Table 3 (Results from analyses on matched cohort 1).**

Maternal and neonatal outcomes in MCoC vs Standard care, with association measures by risk ratios or median difference.

**S. Table 4.**

Summary of associations of preterm birth with MCoC based on Matched cohort 2, *stratified* on parity (No. 2 and 3) as well, and the Hospital-based cohort before matching. E-value was separately reported from sensitivity analysis to *unmeasured* or *uncontrolled confounders*.

**Suppl. Table 1.** Diagnostic codes used in this study to identify cases of outcomes.

| **Clinical Diagnosis** | **ICD-10-SE Code or Procedure code** |
| --- | --- |
| Epidural anesthesia | ZXH50, SN999 |
| Spinal anesthesia | ZXH40 |
| Induction * | O61, O75.6B |
| Elective caesarean without medical indication | O828, Z918 |
| Episiotomy * | TMA00 |
| Intrapartum Oxytocin * | DT037 |
| ^*^Identification by both checkbox and corresponding ICD-10 codes or procedure code. | |

| **Suppl. Table 2 (Results from analyses on matched cohort 1).** Checking on covariate balance (after matching *) across MCoC and standard care groups, indicated by absolute standardized difference. | | | |
| --- | --- | --- | --- |
|  | | | |
| **Characteristic, N (%)** | **MCoC (921 women)** | **Matched standard care (1,823 women)** | **Absolute standardized difference (< 0.1)** |
| **Age, y, mean (SD)** | 33.5 (3.8) | 33.4 (4.2) | 0.007 |
| 15-24 | 10 (1.1%) | 23 (1.3%) | 0.019 |
| 25-29 | 160 (17.4%) | 317 (17.4%) |  |
| 30-34 | 440 (47.8%) | 861 (47.2%) |  |
| ≥35 | 311 (33.8%) | 622 (34.1%) |  |
|  |  |  |  |
| **Height, cm, mean (SD)** | **167.7 (6.6)** | **167.8 (6.3)** | 0.012 |
| ≤ 159 | 83 (9.0%) | 155 (8.5%) | 0.044 |
| 160-164 | 206 (22.4%) | 393 (21.6%) |  |
| 165-169 | 249 (27.0%) | 492 (27.0%) |  |
| ≥ 170 | 368 (40.0%) | 759 (41.6%) |  |
| Unknown | 15 (1.6%) | 24 (1.3%) |  |
|  |  |  |  |
| **BMI, kg/m2, mean (SD)** | **24.0 (4.1)** | **24.2 (4.1)** | 0.043 |
| Underweight | 17 (1.8%) | 31 (1.7%) | 0.059 |
| Normal weight | 583 (63.3%) | 1174 (64.4%) |  |
| Over weight | 207 (22.5%) | 422 (23.1%) |  |
| Obese | 78 (8.5%) | 141 (7.7%) |  |
| Unknown | 36 (3.9%) | 55 (3.0%) |  |
|  |  |  |  |
| **Parity** |  |  | 0.037 |
| Primiparity | 509 (55.3%) | 974 (53.4%) |  |
| Multiparity | 412 (44.7%) | 849 (46.6%) |  |
|  |  |  |  |
| **Multiple pregnancy** | **7 (0.8%)** | **13 (0.7%)** | 0.005 |
|  |  |  |  |
| **Psychiatric care** |  |  | 0.069 |
| Yes | 302 (32.8%) | 563 (30.9%) |  |
| No | 592 (64.3%) | 1221 (67.0%) |  |
| Unknown | 27 (2.9%) | 39 (2.1%) |  |
|  |  |  |  |
| **Level of education, y** |  |  | 0.053 |
| ≤ 9 | 5 (0.5%) | 4 (0.2%) |  |
| 10-12 | 115 (12.5%) | 230 (12.6%) |  |
| > 12 | 749 (81.3%) | 1487 (81.6%) |  |
| Unknown | 52 (5.6%) | 102 (5.6%) |  |
|  |  |  |  |
| **Mother's birth region** |  |  | 0.046 |
| Nordic | 779 (84.6%) | 1564 (85.8%) |  |
| Europe (non-Nordic) | 52 (5.6%) | 92 (5.0%) |  |
| Middle East/Africa | 26 (2.8%) | 46 (2.5%) |  |
| Others | 51 (5.5%) | 91 (5.0%) |  |
| Unknown | 13 (1.4%) | 30 (1.6%) |  |
|  |  |  |  |
| **Given birth during Covid-19 period (since 202003)** | 644 (69.9%) | 1288 (70.7%) | 0.016 |
|  |  |  |  |
| **Pre-pregnancy comorbidity**** | |  | 0.043 |
| Yes | 421 (45.7%) | 835 (45.8%) |  |
| No | 480 (52.1%) | 959 (52.6%) |  |
| Unknown | 20 (2.2%) | 29 (1.6%) |  |
|  |  |  |  |
| **Smoking status at first antenatal care** |  |  | 0.084 |
| Smoker | 7 (0.8%) | 12 (0.7%) |  |
| Non-smoker | 850 (92.3%) | 1720 (94.3%) |  |
| Unknown | 64 (6.9%) | 91 (5.0%) |  |
|  |  |  |  |
| **Previous Caesarean** | 49 (5.3%) | 99 (5.4%) | 0.005 |
|  |  |  |  |
| Abbreviations: MCoC, Midwifery Continuity of Care  *Age, height, and BMI in categorical forms used for in propensity score calculation.  **Pre-pregnancy comorbidity, including cardiovascular disease, liver disease, diabetes, gynecological disease, lung disease, endocrine disease, kidney disease, inflammatory bowel disease, chronic hypertension and neurological disorder | | | |
|  | | | |

**Suppl. Table 3 (Results from analyses on matched cohort 1).** Maternal and neonatal outcomes in MCoC vs Standard care, with association measures by risk ratios.

| **Outcomes** | **MCoC (921 women)** | **Matched standard care (1,823 women)** | **Risk Ratio (95% CI)** |
| --- | --- | --- | --- |
| **Maternal outcomes,** N (%) | | | |
| Elective Caesarean (WMI) | 13 (1.4%) | 68 (3.7%) | 0.38 (0.21, 0.68) |
| Elective Caesarean (WOMI) | 7 (0.8%) | 78 (4.3%) | 0.18 (0.08, 0.38) |
| Emergency Caesarean | 109 (11.8%) | 229 (12.6%) | 0.94 (0.76, 1.17) |
| Spontaneous VD | 745 (80.9%) | 1352 (74.2%) | 1.09 (1.05, 1.13) |
| Instrumental VD | 47 (5.1%) | 96 (5.3%) | 0.97 (0.69, 1.36) |
| Rupture grade III or IV * | 25 (3.2%) | 52 (3.6%) | 0.88 (0.55, 1.42) |
| Total blood loss ≥1000 ml | 86 (9.4%) | 172 (9.5%) | 0.99 (0.77, 1.28) |
| **Labor management,** N (%) | | | |
| Induction | 183 (19.9%) | 426 (23.4%) | 0.85 (0.73, 0.99) |
| Epidural/spinal analgesia ** | 404 (44.8%) | 936 (55.8%) | 0.80 (0.74, 0.87) |
| Intrapartum Oxytocin ** | 339 (37.6%) | 707 (42.2%) | 0.89 (0.81, 0.98) |
| Episiotomy * | 15 (1.9%) | 48 (3.3%) | 0.57 (0.33, 0.98) |
| Amniotomy | 319 (34.6%) | 712 (39.1%) | 0.89 (0.80, 0.98) |
| **Neonatal outcomes,** N (%) | | | |
| Preterm *** | 27 (2.9%) | 122 (6.7%) | 0.42 (0.27, 0.65) |
| Post-term (≥42 weeks) | 29 (3.1%) | 67 (3.6%) | 0.86 (0.56, 1.31) |
| Small for gestational age | 23 (2.5%) | 61 (3.3%) | 0.75 (0.46, 1.20) |
| Large for gestational age | 22 (2.4%) | 61 (3.3%) | 0.71 (0.44, 1.15) |
| Apgar at 5 min < 7 *** | 13 (1.4%) | 32 (1.8%) | 0.80 (0.42, 1.52) |
| Apgar at 5 min < 4 *** | 4 (0.4%) | 8 (0.4%) | 0.98 (0.30, 3.26) |
|  |  |  |  |
| Stillbirth | 0 | 9 (0.49%) | - |
|  |  |  |  |

Abbreviations; MCoC, Midwifery Continuity of Care; WMI, with medical indication; WOMI, without medical indication; VD, vaginal delivery.

**^*^** Association among vaginal delivery, without adjusting for covariates.

**^**^** Association among spontaneous onset and induced labours without adjusting for covariates.

**^***^**Association among live births, without adjusting for covariates.

**Suppl. Table 4.** Summary of associations of preterm birth with MCoC based on Matched cohort 2, *stratified* on parity (No. 2 and 3) as well, and the Hospital-based cohort **^*^** before matching. E-value was separately reported from sensitivity analysis to *unmeasured* or *uncontrolled confounders*.

| **No.** | **Outcomes, N (%)** | **MCoC** | **Matched standard care** | **Risk Ratio (95% CI)** | **E-value ^**^** | **Notes** |
| --- | --- | --- | --- | --- | --- | --- |
| **After propensity score matching (among live births)** | | | | | | |
| 1 | Preterm birth, overall | 23 (3.0) | 113 (5.1) | 0.51 (0.32, 0.82) | E=3.33 for RR  E=1.74 for CI | Matched cohort |
| 2 | Preterm birth, **nulliparous women** | 16 (3.8) | 79 (6.8) | 0.51 (0.29, 0.88) | E=3.33 for RR  E=1.53 for CI | Subgroup |
| 3 | Preterm birth, **multiparous women** | 7 (2.0) | 34 (3.3) | 0.58 (0.26, 1.28) | 1 | Subgroup |
| **Before propensity score matching (among live births)** | | | | | | |
| 4 | Preterm birth, whole study population **^***^** | 28 (2.9) | 860 (7.6) | 0.43 (0.29, 0.65) | E=4.08 for RR  E=2.45 for CI | Hospital-based cohort |

Abbreviations; MCoC, Midwifery Continuity of Care

**^*^** Propensity score matching approaches 1-3 aimed to estimate the effect of MCoC among those in the MCoC Group, comparing to matched standard care (or in a counterfactual setting where they did not receive MCoC), which can be generalized to those with the similar baseline characteristics among any population. In subgroup analyses, covariates multiple pregnancy and mother’s birth country were adjusted for again through multivariable regression (due to the violence of covariate balance after stratification on parous women), similarly age and mother’s birth country were adjusted for among nulliparous women.

** In addition to matching covariates in this study, we considered the potential effect of genetic factors of preterm birth e.g. family history of preterm birth and maternal preterm birth [ref 1], and reported E-values from sensitivity analyses to unmeasured or uncontrolled confounders [ref 2]. E.g. the observed risk ratio of 0.51 with the upper confidence limit of 0.82 could be explained away by an unmeasured or uncontrolled confounder that was associated with both caseload care and preterm birth by risk ratios of 1.74-fold each, but weaker confounding could not potentially move the upper confidence limit to include 1.

******* By the whole hospital-based study samples over the study period, as a naive analysis for extra information, traditional multivariable regression analysis 4 aimed to explore the association between MCoC and preterm births, with adjustment for the same covariates used for propensity score matching. However, the results might not be generalized to other hospitals, which might be due to unbalanced maternal baseline characteristics between hospitals (or due to potential selection bias).

**Reference:**

1. Urquia, Marcelo L., et al. "Revisiting the association between maternal and offspring preterm birth using a sibling design." *BMC Pregnancy and Childbirth* 19.1 (2019): 1-9.
2. VanderWeele, Tyler J., and Peng Ding. "Sensitivity analysis in observational research: introducing the E-value." *Annals of internal medicine* 167.4 (2017): 268-274.
